# Exploring Australian Hajj Tour Operators’ Knowledge and Practices Regarding Pilgrims’ Health Risks: A Qualitative Study

**DOI:** 10.2196/10960

**Published:** 2019-05-23

**Authors:** Amani S Alqahtani, Mohamed Tashani, Anita E Heywood, Robert Booy, Harunor Rashid, Kerrie E Wiley

**Affiliations:** 1 Saudi Food and Drug Authority Riyadh Saudi Arabia; 2 Child and Adolescent Health Children’s Hospital at Westmead Clinical School University of Sydney Sydney Australia; 3 University of New South Wales Sydney Australia; 4 University of Sydney Sydney Australia

**Keywords:** Hajj, health advice, mass gathering, travel agents, travel, respiratory infections

## Abstract

**Background:**

Travel agents are known to be one of the main sources of health information for pilgrims, and their advice is associated with positive health behaviors.

**Objective:**

This study aimed to investigate travel agents’ health knowledge, what health advice they provide to the pilgrims, and their sources of health information.

**Methods:**

In-depth interviews were conducted among specialist Hajj travel agents in Sydney, Australia. Thematic analysis was undertaken.

**Results:**

Of the 13 accredited Hajj travel agents, 9 (69%) were interviewed. A high level of awareness regarding gastrointestinal infections, standard hygiene methods, and the risk of injury was noted among the participants and was included in advice provided to pilgrims. However, very limited knowledge and provision of advice about the risk of respiratory infections was identified. Knowledge of the compulsory meningococcal vaccine was high, and all participated travel agents reported influenza vaccine (a recommended vaccine) as a second “compulsory” vaccine for Hajj visas. Conversely, participants reported very limited knowledge about other recommended vaccines for Hajj. The Ministry of Hajj website and personal Hajj experience were the main sources of information.

**Conclusions:**

This study identifies a potential path for novel health promotion strategies to improve health knowledge among Hajj travel agents and subsequently among Hajj pilgrims.

## Introduction

As the world’s largest annual mass gathering, Hajj presents a major challenge to disease control in Saudi Arabia, as more than 2 million people travel each year to commemorate the pilgrimage [1]. This challenge is not only for Saudi Arabia but is also international with the risk of pilgrims importing infectious diseases into their home countries when they return [2]. Respiratory tract infections, including influenza and pneumonia, are the leading health risks at Hajj [3-6]. Emerging infections like Middle East respiratory syndrome coronavirus (MERS-CoV) pose additional threats to disease control [7,8]. The Saudi Arabian Ministry of Health (MoH) and World Health Organization have introduced and recommended various preventive health measures for pilgrims including vaccines and other disease control measures (Table 1 and Textbox 1) [9].

**Table 1:** Summary of the recommended vaccines for the prevention of infectious disease in travellers to Saudi Arabia for Hajj.

Preventive measures		Instructions	Remarks
**Compulsory vaccines**			
	Quadrivalent meningococcal vaccine (ACWY)	Compulsory for all pilgrims	Administered not less than 10 days before arrival
	Oral polio (OPV) or inactivated poliovirus (IPV)	Compulsory for pilgrims from endemic countries	Administered at least 4 weeks before arrival Other pilgrims should remain up to date
	Yellow fever vaccine	Compulsory for pilgrims from endemic countries or those transiting through endemic countries	Administered at least 10 days before arrival
**Recommended vaccines**			
	Seasonal influenza vaccine	Recommended for all, in particular at-risk pilgrims	N/A^a^
	Vaccines against diphtheria, tetanus, pertussis, measles, and mumps	Remaining up to date	N/A

^a^N/A: not applicable.

**Summary of the recommended non-pharmaceutical measures for the prevention of infectious disease in travellers to Saudi Arabia for Hajj.**

**Non-pharmaceutical measures**

- Wash hands with soap and water or disinfectant, especially after coughing and sneezing, after using the toilet, before handling and consuming food, and after touching animals
- Use disposable tissues when coughing or sneezing and dispose of them afterwards in waste baskets
- Avoid hand contact with the eyes, nose, and mouth
- Wear facemasks, especially in crowded places
- Avoid direct contact with persons who appear to be ill with coughing, sneezing, expectorating, vomiting, diarrhea, and do not share personal belongings
- Maintain good personal hygiene
- Avoid contact with sick animals
- Avoid drinking raw camel milk or camel urine or eating meat that has not been properly cooked
- Take insect bite avoidance measures during daytime and night time hours to reduce the risk of infection with dengue and other mosquito-borne diseases

**Health education**

- Health authorities in countries of origin are required to provide health information to pilgrims on infectious disease symptoms, transmission mode, and measures to prevent

Travel to Hajj is unique compared to other types of travel, including travel to other mass gathering events, in that pilgrims must travel in groups by enrolling with a travel agent authorized by the Saudi Arabian authority to run Hajj tourism [10]. These tour operators play an important role in preparing pilgrims for their journey, including securing Hajj visas, conducting pre-travel briefing sessions about the ritual steps (popularly known as “Hajj seminars”), and providing some safety advice to pilgrims to enable a safe journey for their clients [10].

The provision of pre-travel health advice plays an important role in raising awareness among the travellers of the health risks and preventive measures. Seeking health advice from travel agents has been reported previously among travellers including Hajj pilgrims [11-15]. Recent quantitative studies have found that Hajj pilgrims report travel agents as one of their main sources of health information, and their advice was associated with increased likelihood of positive travel health behaviors [14,16-21]. However, no studies have attempted to investigate the provision of pre-travel health advice from the travel agents’ perspective. We conducted a qualitative study aimed at addressing this knowledge gap through better understanding travel agents’ knowledge about the health risks at Hajj, what health advice they provide to the pilgrims, and their sources of health knowledge.

## Methods

### Study Design

A qualitative study was conducted to enable exploration of unknown or unanticipated issues not usually possible with quantitative methods. Qualitative methods provide detailed, in-depth insights into why people act the way they do. A grounded theory approach was used [22], as it provided the ideal methodological framework in which to explore the pre-travel health advice Hajj travel agents provide to pilgrims in a way that allows for unexpected findings to be further explored. Grounded theory uses an inductive approach, whereby a cycle of data collection (interviews) and analysis is followed by subsequent interview and analysis cycles. Themes emerge from earlier interviews that inform subsequent interview questions to allow further exploration of emergent themes. This cycle continues until no new themes emerge and theoretical saturation is reached.

### Sampling Strategy and Participants

A purposive sampling strategy was employed. This study was conducted in Sydney, New South Wales (NSW), Australia. Our research group previously conducted quantitative studies in 2014 and 2015 among Sydney-based Hajj pilgrims [14,16-19]. Sampling Sydney-based travel agents enabled us to further examine the quantitative survey findings. Agents were invited to participate in the study through email or telephone. Once the agent responded and agreed to participate, a consent form was sent via email or post, and verbal consent was obtained from all participants prior to interview.

### Interviews

Between February and September 2016, in-depth interviews were conducted. All interviews were audio recorded with the participants’ verbal consent. Participants were assured that interviews would be strictly confidential and any published results would contain non-identifying data only. Each of the interviews took between 30 and 60 minutes to complete. While English was the primary language used in the interviews, participants were given the option to be interviewed in Arabic if they preferred.

A semistructured interview guide was used that evolved with each iteration of the grounded theory data collection and analysis cycle [22]. Participants were encouraged to narrate events and situations they experienced and to describe the circumstances around their behaviors and choices. Participants’ actual behaviors and experiences were the focus, rather than their opinions. The interviews explored the pre-travel health services and information that tour group leaders provide to the pilgrims in regard to the risk of health hazards at Hajj and the preventive measures they promote to protect the pilgrims against those health risks. The interviews also explored pre-travel health advice issues such as access, utilization and barriers to health services, and current information sources and their cultural appropriateness.

### Analysis

Throughout the process, the first author kept a research journal detailing experiences and thoughts in relation to the collection and analysis of the data. This method is known to promote reflexivity on the part of the researcher during data collection, analysis, and reporting. The interviewer independently constructed a code list of major themes emerging from the data using line-by-line coding of the transcripts, followed by focused coding to synthesize emergent themes from the data. Finally, axial coding was used to draw relationships between the themes. The coding of transcripts and the compilation and analysis of code files were done using NVivo 11 software.

### Ethical Approval

This study was reviewed and approved by the Human Research Ethics Committee (HREC) at the University of Sydney (Project No. 2014/599).

## Results

### Participant Characteristics

There are 13 accredited Hajj tour group leaders operating in Sydney, Australia. All were approached, and 9 (69%) agreed to participate in the study. Eight of nine (89%) were male, all were aged between 38 and 65 (median 45) years, and the number of years of Hajj travel business experience among participants ranged between 3 and 30 (median 9) years.

Thematic analysis of interview responses identified two main themes: (1) the type of advice given to pilgrims by these travel agents and (2) the sources of health information for travel agents. Each key theme can be divided into subthemes. In the following sections, the main findings will be described and links between themes highlighted.

### Major Theme 1: The Type of Health Advice Provided to Hajj Pilgrims by Travel Agents

Generally, advice provided by travel agents typically included the compulsory vaccination requirements for securing the Hajj visa, standard hygiene methods such as hand hygiene, and some safety advice to prevent injury or trauma during Hajj. A typical response to the question of what kinds of general advice they provide to pilgrims was:

We usually provide them information regarding the required vaccines for the visa and general hygiene practice such as keeping their hand clean most of the time. We also ask them to follow the group leader all the time so they can perform the Hajj in the best way.

Male, 65 years old

Most of the agents additionally requested at-risk pilgrims (the elderly and/or those with chronic conditions) to provide a letter from their local doctor about their ability to perform Hajj. Also, they advised pilgrims to bring adequate quantities of their regular medications along with prescriptions for these medications. For example:

We also ask the pilgrims to bring a medical certificate from their doctors to approve that he or she is fit physically and able to do the Hajj pilgrimage, and this only for pilgrims who are elderly or have any medical conditions.

Male, 37 years old

Yes, I ask them to bring adequate medication and have a certificate from the doctor that they are able to do the Hajj.

Male, 42 years old

#### Specific Advice About Health Risks During Hajj

When asked what specific advice they give to prospective pilgrims about health risks, the majority of travel agents spontaneously responded with their advice about non-infectious ailments such as sunstroke, trauma and injuries, and food-borne infectious diseases. None mentioned the risk of communicable diseases, with the exception of influenza:

Usually in Hajj, the major things they can face in there is the crowded (sic) and the dehydration. So this is a very major issue because it’s really hot weather and the hot climate (sic) especially for the elderly, and also the food is really, really important because they need to eat healthy, clean food, and need to know where to buy it from, and all that sort of things.

Male, 48 years old

The risks mainly were about the diarrhea, indigestion, and flu. That’s what we usually advise, plus heat stroke.

Male, 37 years old

#### Advice on the Risk of MERS-CoV During Hajj

Few agents were aware of the emerging infection, MERS-CoV, in Saudi Arabia. Of those who knew about it, there was a demonstrated lack of knowledge or incorrect beliefs about issues such as mode of transmission and appropriate preventive measures.

No, we were not aware of MERS-CoV in particular, but we only advised them about the problem with the poor health condition of some third world countries, which causes some serious diseases that do not exist in Australia. So we usually ask them to avoid some crowded places.

Male, 44 years old

Yes, like in 2009 we told them to take care of their selves from H1N1, so in MERS we told them that there is more severe infection now.

Male, 65 years old

Yeah, we heard about it and it’s been, like, there for about two…three years now. So, as I said, we told them that this infection can be transferred, it’s contagious, and could be by blood or by water and also by breath.

Male, 46 years old

Postponement of Hajj travel for at-risk groups was the only preventive measure for MERS-CoV that was reported (by two travel agents). No other advice or recommendations about MERS-CoV preventive measures such as facemasks was reported. Travel agents reported a lack of regular information about MERS-CoV from the Saudi Arabian Embassy as a factor in not providing advice.

In Hajj 2014, the Saudi Embassy sent us a message and advised to visit some links which has some information regarding MERS and advised the elderly to not perform the Hajj, but not last year (2015) or this year (2016). Actually, this was very good and helped us to provide the pilgrims proper information. But this year we did not give them any advice regarding MERS-CoV as we did not receive any message from the Saudi Embassy.

Male, 48 years old

#### Vaccination Advice

The meningococcal vaccine (ACWY) is a compulsory vaccine for obtaining the Hajj visa, and most travel agents described a high level of knowledge regarding meningococcal disease severity and vaccine effectiveness in protecting pilgrims. This knowledge was also shared with the pilgrims as a further incentive to get the vaccine.

We described to them that this disease is fatal, and I also give them examples about myself that I always get this vaccine and renew the certificate when it’s expired. In Australia, this infection is rare, but in some countries this infection maybe common and as Hajj involved many pilgrims from different countries, this may increase the risk to get this disease during Hajj.

Male, 65 years old

Yes, we do. Because we know that it’s a dangerous disease and if they catch it they will be in grave danger of life and so forth. So yes, we do advise them that it’s important that you need to take it.

Male, 37 years old

Other agents described the importance of the meningococcal vaccine only to get the visa and relied on doctors to provide any further medical information when the pilgrims visited them to get the vaccine.

I told them that without this vaccine you cannot get the visa, but medically, we deal with some specific doctors and advise the pilgrims to visit them and they provide them the medical information about the vaccine.

Male, 46 years old

When the travel agents were asked if they recommended any other vaccine to the pilgrims besides meningococcal vaccine, interestingly, all travel agents reported seasonal influenza vaccine as a second “compulsory” vaccine for the visa, despite its being “recommended” and not “required” according to the Saudi Arabian Ministry of Health website.

Yes, I also asked them to have flu vaccine because it is compulsory too.

Male, 55 years old

The flu vaccine is compulsory too.

Male, 39 years old

Yes ACWY, that’s meningococcal and then they need to have a seasonal flu vaccine, so these two are required before they can even be given visa and the medical certificate are attached to the back of the passport.

Male, 44 years old

None of the travel agents recommended any vaccines other than the compulsory one or those they considered compulsory. They considered this to be the doctors’ job as they are medically qualified and have a better understanding of the pilgrim’s health history.

I don’t really recommend any other vaccine. I’m not a doctor myself. But I recommended them to speak to their local doctor and if they would like them to take extra vaccines.

Male, 40 years old

#### Advice on Nonpharmaceutical Measures

Constant attention to hand hygiene was strongly advised among the non-pharmaceutical measures. Advice to use facemasks was also reported among some travel agents, especially in crowded places and peak times. No other recommendations for preventive measures were spontaneously reported among the travel agents.

I always advised them to wash their hands, especially before and after eating and when using the toilet. We also advised them to use facemask, especially in crowded places and peak hours.

Male, 65 years old

Mainly the advice was washing the hands, always keeping washing the hands at certain times like when you go out, come back to your room, before you eat, wash your hands.

Male, 39 years old

Some agents stated that they continually follow their pilgrims during the Hajj pilgrimage and remind them to use hand hygiene and facemasks.

Yes, we usually visit them in the tent especially in Arafat and Mina and, for example, if the weather is hot we advise them to stay inside the tent. And if we will move and if we expect any crowds, we asked them to put on the mask. Also I keep remind them about hand hygiene and food hygiene.

Male, 55 years old

#### Dissemination of Health Advice by Travel Agents

All Hajj travel agents conducted pre-Hajj seminars several weeks before travel to Hajj. While some discussion was included, there was no specific health session conducted as a part of these seminars.

We organize Hajj seminars to teach them the Hajj worship; sometime we shared some health advice as well.

Male, 42 years old

Well, in our general booklet, we do talk about some issues of health, but it’s not a particular book in just specifically for health.

Male, 37 years old

### Major Theme 2: Sources of Health Information for Travel Agents

Travel agents described obtaining health information from different sources. The Ministry of Hajj and Umrah website was the main cited source.

Mostly I sought the information from Ministry of Hajj website (Saudi Arabia), I do not use any other sources; I only give information of what Ministry of Hajj advises.

Male, 48 years old

Yes, Ministry of Hajj Saudi Arabia, we just do not use any other source, because we have no other information to be verified. So we just only give information of what Ministry of Hajj advises.

Male, 44 years old

Moreover, they also relied on their previous Hajj experiences as a source of their health information.

My health information is mostly from my Hajj experience. I have been organizing the Hajj travel for about 30 years.

Male, 65 years old

Only one agent mentioned the Australian government website, Smartraveller. This travel agent found this website to be disappointing because it does not provide enough information regarding Hajj, so they do not visit it frequently.

Smartraveller has no information apart from threatening the people to not go there, do not talk to this, and do not do this; it’s useless source. They are not culturally aware and they just only give negative information. So it’s not sufficient at all.

Male, 37 years old

Interestingly, none of the participants mentioned the Saudi Arabian MoH website (different to the Ministry of Hajj site mentioned above) as a source of their health information. When asked if they referred their pilgrims to visit any specific Hajj website, some agents encouraged their clients to visit the general Hajj websites but not the Ministry of Health website.

Usually yes, and once we send them information via email there’s a link down there of some websites about Hajj, so they can retrieve it at any time.

Male, 44 years old

Yes, we advised them if they need any further information to Google some Hajj websites.

Female, 45 years old

None of the travel agents undertook any special pre-Hajj training program either as a requirement for approval from the Saudi Arabian authority or just to update their knowledge of health regulations regarding Hajj.

No, we aren’t required to have those sorts of programs and I think it must be included. So for me being a younger Hajj group leader, I feel it’s a duty of care in all this so I always seek the information from different sources. But there’s a lot of older Hajj group organizers that pretty much do not have the resources that I have. I’m not aware if the Ministry of Health, or Ministry of Hajj have such programs for this.

Male, 44 years old

#### Type of Health Information Hajj Pilgrims Usually Seek From the Travel Agent

Travel agents stated that, in their experience, information regarding the visa requirements, weather, and crowding during Hajj was the most sought after by pilgrims. Among those who take regular medications, most inquiries were about the way they can carry their medications and keep them in good condition during their travel.

They were asking about vaccines for the visa, the crowd, or the general conditions and the temperature and also the travelling route, the toilet, and these are just general basic handling that they need to look at what they require during their travel. That’s all they’re usually worried about.

Male, 37 years old

Mostly those who have diabetes and asthma, they’re concerned and always asked about their medication, how they can bring it with them and keep it safe in the journey. As you know, specially the diabetes medication it should be kept cool, so we provide the cold container when we travel from Australia to Saudi Arabia and from Madinah to Makkah.

Male, 55 years old

#### Barriers to and Facilitators of the Availability of Health Information

All respondents reported that there were no barriers regarding health information access because much of the information is widely available through the Internet. The travel agents assumed the information they provide to their clients (Hajj pilgrims) is adequate to make the pilgrims’ travel safe.

Well, this generation is more aware and knowledgeable; they always visit the Internet to get all the information they need.

Male, 65 years old

I don’t think there is a lack of information. It’s alright. We’ve given them all the information they need.

Male, 48 years old

Travel agents suggested some methods to better deliver health information to Hajj pilgrims. This included providing health lectures for Australian pilgrims before travel, conducted by Muslim doctors; disseminating health booklets organized by the Saudi health authority at entry points to Saudi Arabia or through the Saudi embassies in pilgrims’ original countries; and supplying the travel agents with adequate, factual health information so they can pass it on to pilgrims.

All the information is accessible; however, if the Saudi Ministry of Health or Ministry of Hajj provide us a booklet that have (sic) all the health information regarding the vaccinations and any other measures that the pilgrims need, this will be a better way to deliver the information, so the pilgrim can keep it with them during Hajj journey. Like what happened in 2014, they provided us booklets which were very helpful. Or provide it in the entry point in Mecca or Medina but better to be before Hajj so they can well prepare.

Male, 65 years old

### The Relationships Between the Identified Themes

The policy system that develops Hajj health regulations is under the “umbrella” of the Saudi Ministry of Health and Ministry of Hajj, with the involvement of local health authorities in the original country of pilgrims [9,10]. However, personal Hajj experience and the Ministry of Hajj website were the most direct influencers of health knowledge among the interviewed Hajj tour operators. Other indirect factors such as Hajj experiences of their previous clients and advice from the Saudi Arabian Embassy were also influential. Health information was distributed through the pre-Hajj seminars run by the tour operators, either verbally or in written form (Multimedia Appendix 1).

## Discussion

### Principal Findings

This study provides insight into Hajj travel agents’ knowledge and awareness of health issues and the health advice they provide to their Hajj travellers. It demonstrates that advice from travel agents was the main source of knowledge about vaccines and significantly improved vaccine uptake, compared to advice from other sources such as general practitioners, in Hajj 2014 and 2015 [14]. It also illustrates that, with the exception of influenza, the agents reported very limited knowledge and advice about the risk of respiratory infectious diseases, which are the most common health hazards at Hajj [3,23].

This study found a high level of knowledge among travel agents regarding non-infectious illnesses such as sunstroke, trauma and injuries, and food-borne infectious diseases. These results agree with the findings of our previous quantitative studies where we found that Australian Hajj pilgrims in both years (2014 and 2015) were very concerned about the risk of food-borne diseases, diarrhea, influenza, and trauma [14], yet showed a lower level of concern about other common respiratory infections at Hajj, including pneumonia. Interestingly, in this study, while few tour operators were aware of the ongoing MERS-CoV risks in Saudi Arabia and had limited knowledge about its transmission mode and preventive measures, some participants mentioned that the advice about MERS-CoV was distributed only in 2014, but not in 2015 and 2016. Information from the Saudi Arabian Embassy in 2014 (that we deem appropriate) was the main source of their knowledge. These results align with the pilgrim knowledge base examined in our quantitative surveys, which found a decline in pilgrims’ awareness about MERS-CoV in 2015 (117/421, 27.8%) compared with the previous survey in 2014 (179/350, 51.1%) [16,17]. Given that no cases were detected at these Hajj events, education regarding MERS-CoV was arguably adequate.

Unsurprisingly, in this study we found that knowledge among travel agents about the compulsory meningococcal vaccine was high, including the severity of the disease and the transmission mode. Remarkably, all participating travel agents also reported seasonal influenza vaccine as a second compulsory vaccine for obtaining the Hajj visa. This helps explain the finding from the quantitative surveys of pilgrims, which reported a comparatively high rate of influenza vaccine coverage among Australian Hajj pilgrims in several years (80% in 2014 [14] and 76% in 2015), which is much higher than for other countries (eg, 7.1% among Turkish pilgrims in 2015 [24] and 20% among Egyptian pilgrims between 2012 and 2015 [25]). In addition, our qualitative data revealed no knowledge or advice about the other recommended vaccines from the sampled Hajj travel agents. This result aligns with the quantitative findings relating to the low uptake of other recommended vaccines such as pneumococcal and pertussis vaccines among Australian Hajj pilgrims [14]. Similarly, low uptake has also been reported among international Hajj pilgrims [26].

While our study found that no pre-Hajj training programs have been offered to the Hajj tour operators, , this study showed that the Ministry of Hajj and Umrah website and personal Hajj experience were the main sources of information for the advice travel agents provided. This is an important finding given the fact that the Ministry of Hajj and Umrah is mostly responsible for the management of administrative procedures of Hajj and Umrah, not for ensuring the health of Hajj pilgrims. The website provides information for authorized Hajj coordinators about related services such as issuing visas and organizing accommodation. Although the Saudi Arabian MoH is the official source of information about the health conditions for Hajj travellers, none of the sampled travel agents reported it as a source of their health knowledge. This also aligns with findings from the quantitative survey, which found more than half of Australian pilgrims were not aware of the official health recommendations issued by the Saudi Arabian MoH [14]. This may indicate that published official guidelines do not uniformly reach the travel agents, let alone reach the Hajj pilgrims.

Another important stakeholder identified from this study is the Saudi Arabian Embassy in Australia. This study found that information distributed from the embassy to the authorized Hajj travel agents helped increase the agents’ knowledge regarding MERS-CoV in 2014. The Saudi Arabian Embassy is considered the first contact point for organizing Hajj travel including providing the approval for travel agents to conduct Hajj travel and receiving the applications for Hajj visas [10]. This unique finding identifies a potential path for a new health promotion strategy to help improve the health knowledge among Hajj travel agents and, as a result, among Hajj pilgrims. This can be achieved by encouraging the Saudi embassies around the world to disseminate accurate and adequate health information from the MoH to all authorised Hajj travel operators, and provide annual pre-Hajj training and educational programs to the tour operators, encouraging them to distribute the information to their pilgrims. It is noteworthy to highlight that the current health guidelines for Hajj travellers are published in medical journals or other platforms primarily targeted for health care providers, using technical/medical language that would be difficult for lay tour operator or pilgrims to comprehend. There is a need for a guideline published in simple and understandable language for lay pilgrims and tour operators [27].

### Limitations

To our knowledge, this is the first qualitative study involving Hajj tour operators. It provides valuable insight into travel agents’ knowledge, attitudes, and advice related to health risks and preventive measures during Hajj. Nevertheless, this study has some limitations. While our purposive sample included 70% of the Hajj travel agents in NSW, who service the Hajj travel needs of 49% of Australia’s Muslims, the inclusion of Hajj travel agents from other states and territories might have yielded themes not identified here. Time and resource constraints resulted in recruitment ceasing at nine participants, meaning that most, but not all, themes met theoretical saturation. Another limitation was that only the first author was involved in coding the transcripts. This may limit the credibility of the coding, although other senior authors were involved in the discussion and development of the thematic analysis throughout the process.

### Conclusion

This study identifies important opportunities to use Hajj travel agents and Saudi embassies as conduits for health promotion for Hajj pilgrims and can inform travel health policy and practice.

## Appendix

Multimedia Appendix 1 Factors influencing the health knowledge and practices of Hajj tour operators.

## Abbreviations

ACWY: quadrivalent meningococcal vaccine
MERS-CoV: Middle East respiratory syndrome coronavirus
MoH: Ministry of Health (Saudi Arabia)
NSW: New South Wales
