# Supplementary material for: Exploring Australian Hajj Tour Operators’ Knowledge and Practices Regarding Pilgrims’ Health Risks: A Qualitative Study
Source: JMIR Public Health Surveill. 2019 May 23;5(2):e10960. doi: 10.2196/10960 (PMC6552451; doi:10.2196/10960)
Supplement: Multimedia Appendix 1 [file publichealth_v5i2e10960_app1.doc]

Multimedia Appendix 1. Factors influencing the health knowledge and practices of Hajj tour operators.
